# An In Vitro Barrier Model of the Human Submandibular Salivary Gland Epithelium Based on a Single Cell Clone of Cell Line HTB-41: Establishment and Application for Biomarker Transport Studies

**DOI:** 10.3390/biomedicines8090302

**Published:** 2020-08-23

**Authors:** Grace C. Lin, Merima Smajlhodzic, Anna-Maria Bandian, Heinz-Peter Friedl, Tamara Leitgeb, Sabrina Oerter, Kerstin Stadler, Ulrich Giese, Johannes R. Peham, Lynne Bingle, Winfried Neuhaus

**Affiliations:** 1 Competence Unit Molecular Diagnostics, Center for Health and Bioresources, Austrian Institute of Technology (AIT) GmbH, Giefinggasse 4, 1210 Vienna, Austria; Grace.Lin@ait.ac.at (G.C.L.); m.smajlhodzic@gmail.com (M.S.); anna.bandian@gmail.com (A.-M.B.); Heinz-Peter.Friedl@ait.ac.at (H.-P.F.); tl.leitgeb@gmail.com (T.L.); stadler.kers90@gmail.com (K.S.); giese.u@googlemail.com (U.G.); johannes.peham@ait.ac.at (J.R.P.); 2 Fraunhofer Institute for Silicate Research (ISC), Neunerplatz 2, 97082 Würzburg, Germany; sabrina.oerter@uni-wuerzburg.de; 3 School of Clinical Dentistry, University of Sheffield, Claremont Crescent, Sheffield S10 2TA, UK; l.bingle@sheffield.ac.uk

**Keywords:** salivary gland, submandibular, blood–saliva barrier, Sjögren’s syndrome, rheumatoid arthritis, periodontitis

## Abstract

The blood–saliva barrier (BSB) consists of the sum of the epithelial cell layers of the oral mucosa and salivary glands. In vitro models of the BSB are inevitable to investigate and understand the transport of salivary biomarkers from blood to saliva. Up to now, standardized, cell line-based models of the epithelium of the submandibular salivary gland are still missing for this purpose. Therefore, we established epithelial barrier models of the submandibular gland derived from human cell line HTB-41 (A-253). Single clone isolation resulted in five different clones (B2, B4, B9, D3, and F11). Clones were compared to the parental cell line HTB-41 using measurements of the transepithelial electrical resistance (TEER), paracellular marker permeability assays and analysis of marker expression for acinar, ductal, and myoepithelial cells. Two clones (B9, D3) were characterized to be of acinar origin, one clone (F11) to be of myoepithelial origin and one isolation (B4) derived from two cells, to be presumably a mixture of acinar and ductal origin. Clone B2, presumably of ductal origin, showed a significantly higher paracellular barrier compared to other clones and parental HTB-41. The distinct molecular identity of clone B2 was confirmed by immunofluorescent staining, qPCR, and flow cytometry. Experiments with ferritin, a biomarker for iron storage, demonstrated the applicability of the selected model based on clone B2 for transport studies. In conclusion, five different clones originating from the submandibular gland cell line HTB-41 were successfully characterized and established as epithelial barrier models. Studies with the model based on the tightest clone B2 confirmed its suitability for transport studies in biomarker research.

## 1. Introduction

Since saliva offers the possibility of non-invasive sample collection accompanied with minimal contamination risk, efforts have been made to validate salivary biomarkers in the past decades. Up to now, several salivary biomarkers for cancer, infectious, or autoimmune diseases are already in use [1,2,3,4]. For the assessment of the relevance of a salivary biomarker it is inevitable to know its origin in the body and how the biomarker came into saliva. While the correlation between concentrations of specific salivary biomarkers in serum and saliva is well understood, in-depth knowledge about the appearance of those molecules in saliva is still missing. In 2018, Bierbaumer et al. defined the blood–saliva barrier (BSB) as the sum of epithelial cell layers from the oral mucosa and salivary glands. Salivary biomarkers derived from blood have to cross the BSB [5]. In general, there are three major salivary glands in the human body producing either serous or mucous saliva. The largest salivary gland, the parotid gland, is known to produce mainly serous saliva, while the submandibular gland produces a mixture of serous and mucous saliva and the sublingual gland, the smallest major salivary gland, produces mostly mucous saliva [6,7]. Acini, clusters of epithelial cells, are responsible for the production of saliva, which is then transported through intercalated ducts to striated ducts entering the oral cavity. Myoepithelial cells envelop the acinar and ductal cell structure and are known to play a role in differentiation of the epithelial cells by, e.g., secretion of growth factors. By contraction of myoepithelial cells upon stimulation of salivary glands to secrete saliva, they also facilitate the transport of saliva and prevent damage of other cells [8,9,10]. Typical cellular markers for myoepithelial cells are vimentin or cytokeratin (CK) 14, while for ductal cells CK7 was described as marker in literature previously [11,12,13]. For acinar cells α-amylase was reported as a marker, whereas expression of CK18 was found in acinar as well as in ductal cells [12,14].

While a significant number of cell line based in vitro models for the oral mucosa epithelium have already been described, only a few salivary gland epithelium models were reported, and most of them are based on cells from the largest major salivary gland, namely the parotid gland.

In this study, we focused on the establishment of human in vitro models for the submandibular salivary gland. Up to now, most studies with submandibular salivary gland models used rat (SMG-C6, SMIE) or mice (CSG 120/7) derived cell lines [15,16,17,18]. These models were applied for mechanistic studies—e.g., the effect of adiponectin receptors on secretion of saliva or the influence of insulin-like growth factor-1 or TNF- α (tumor necrosis factor) on epithelial barrier properties—but not for biomarker or drug transport studies.

Even though models of salivary glands based on primary human cells have been described to form a distinct paracellular barrier previously, the restricted availability of biopsies of salivary glands limit their applicability. Moreover, donor variations also result in decreased reproducibility of the experiments [19]. Hence, for standardized transport studies a thoroughly characterized model based on a human cell line seems to be advantageous. In this context, a distinct paracellular barrier is important to recapitulate tightness properties of the salivary gland epithelium. Moreover, strong tight junctions are also a prerequisite for the correct localization or polarization of receptors and transporter proteins mediating transcellular transport processes [20].

Here, we describe the ability of the submandibular salivary gland epithelium cell line HTB-41 (A-253) to form a significant paracellular barrier. Single cell cloning resulted in further optimization of the model. Transport studies with ferritin across the optimized HTB-41 model were compared to studies with a human oral mucosa model based on cell line TR146 and confirmed the feasibility of these models for salivary biomarker assessment.

## 2. Experimental Section

### 2.1. Cell Culture

The submandibular salivary gland cell line HTB-41, first isolated and described in 1973 by Giard et al. [21], was purchased from ATCC and cultivated in McCoy’s 5A media (Thermo Fisher, Waltham, MA, USA; 16600-082) supplemented with 1% Pen/Strep (Penicillin/Streptomycin, Merck, Darmstadt, Germany; A2213) and 10% FCS (Fetal Calf Serum, Sigma-Aldrich, St. Louis, MO, USA; F9665), in the course of this manuscript termed as McCoy media. The buccal oral mucosa cell line TR146 was purchased from Sigma-Aldrich and cultivated in Dulbecco’s modified Eagle medium (DMEM, Sigma-Aldrich, St. Louis, MO, USA; D5796) supplemented with 1% Pen/Strep and 10% FCS, termed as DMEM media. Cells were detached with 0.05% trypsin/0.02% EDTA (Merck, Darmstadt, Germany; L2143), seeded with a cell density of 8 × 10^3^ cells/cm^2^ for HTB-41 or 9.33 × 10^3^ cells/cm^2^ for TR146 in 5 mL medium in T25 TC-treated cell culture flasks (Greiner Bio-One GmbH, Kremsmünster, Austria; 690175) once a week and cultivated at 37 °C, 5% CO_2_, 95% air atmosphere and 95% humidity. Media change was performed every 2–3 days.

#### Single Cloning of HTB-41 Cells

To isolate single clones from cell line HTB-41, transparent 96-well plates (Greiner Bio-One GmbH, Kremsmünster, Austria; 655180) were coated with 50 µL 0.5% (*w*/*v*) gelatin (SERVA, Electrophoresis GmbH, Heidelberg, Germany; 22151.02) in H_2_O bidest. per well for 30 min at room temperature prior to seeding. Cell seeding was performed with a cell concentration of 10 cells/mL and 20 cells/mL (100 µL/well) at passage 8. Conditioned media from T25 flasks with parental HTB-41 cells was collected with every media change of the flasks and stored at 4 °C until usage. Media change of 96-well plates for single cloning was performed with conditioned media diluted 1:2 with fresh McCoy media. As soon as the clones from the wells B2, B4, B9, D3, and F11 reached confluency in the 96-well plates, they were propagated on 24-well plates (Greiner Bio-One GmbH, Kremsmünster, Austria; 662160) pre-coated with 0.5% gelatin (300 µL/well) and were further cultivated with media change every 2–3 days using fresh McCoy media. Cells were transferred to T25 flasks after reaching confluency on 24-well plates and propagated using the cell seeding density as described above.

### 2.2. Transwell Studies

For transepithelial electrical resistance (TEER) experiments HTB-41 as well as isolated clones were seeded at a cell density of 8 × 10^4^ cells/cm^2^ in 300 µL media in the apical compartment of 24-well Transwell^®^ inserts (Greiner Bio-One GmbH, Kremsmünster, Austria; 662641), whereby 900 µL media was provided in the basolateral wells. Parental HTB-41 cells were seeded in McCoy media at passages 5–12, isolated clones from cell line HTB-41 were seeded at passage 5–12 after single cell cloning at passage 8. Seeding procedure of TR146 was described in detail previously [22].

As soon as cells reached confluency on the Transwell^®^ inserts, cultivation condition was either switched to airlift for designated inserts or stayed under submerged condition. For inserts cultivated under airlift conditions, apical media was added for TEER measurements and removed after the measurement. TEER measurement was performed after media change using the Milicell^®^ ERS-1 Voltohmeter (Merck, Darmstadt, Germany; MERS00001). The chopstick electrode (World Precision Instruments, Sarasota, FL, USA; STX2) was sterilized for a maximum of 10 min in 70% sterile EtOH and incubated in McCoy media for at least 10 min before the measurement. The cells were equilibrated at room temperature for at least 30 min prior to TEER measurement. The output of the measurement (Ω) was multiplied with the surface area of the Transwell^®^ insert after subtraction of the mean value of three blank inserts.

Upon reaching maximum TEER values, the permeability assay using the paracellular marker carboxyfluorescein was performed. For this, apical media was replaced with media containing 10 µM carboxyfluorescein (Sigma-Aldrich, St. Louis, MO, USA; 21877). Cells were incubated for 2 h in the dark at 37 °C in the incubator. Samples were collected and stored at 4 °C in the dark until measurement. Fluorescence of carboxyfluorescein samples was measured at 488 nm excitation/520 nm emission wavelength using an Enspire Multimode Plate Reader (PerkinElmer, Waltham, MA, US). The permeability coefficient (µm/min) was calculated according to Neuhaus et al. (2008) [23].

### 2.3. Characterization of HTB-41 Clones with Cell Type-Specific Markers

For the characterization of the parental cell line and the clones claudin-1 (CLDN1), E-cadherin (E-cad), and *Zonula occludens-1* (ZO-1) were selected as markers for the submandibular gland epithelium [24,25,26] and α-amylase (AMY) as acinar marker [11]. Additionally, the expression of cytokeratin 18 (CK18) was described in acinar and ductal cells previously [12,14,27]. As myoepithelial markers cytokeratin 14 (CK14) and vimentin (Vim) were selected [11,12,13,14]. Cytokeratin 7 (CK7) was predominantly described as a ductal cell marker, while S100A4 was classified as ductal or myoepithelial marker according to literature [12,13,14,25,28], shown in Table 1.

#### 2.3.1. Quantitative Real-Time PCR (qPCR)

Two 24-well Transwell^®^ inserts were lysed with 350 µL lysis buffer (RA1 buffer (Machery-Nagel, Düren, Germany; 740961) supplemented with 1% β-mercaptoethanol) and pooled together as one biological sample. RNA was isolated using the NucleoSpin RNA kit (Machery-Nagel, Düren, Germany; 740955.250) according to the manufacturer’s instruction and each sample was eluted with 40 µL nuclease-free water (Invitrogen, Carlsbad, CA, USA; AM9937). For cDNA synthesis with the Multiscribe Reverse Transcriptase Kit (Thermo Fisher, Waltham, MA, USA; 4311235) 350, 650, 700, or 1000 ng RNA were applied to synthesize 20 µL cDNA solution. QPCR was performed in white 96-well plates (4titue, Dorking, UK; 4ti-0951) with 20 µL reaction volume containing 4 µL diluted (1:3.5, 1:6.5, 1:7, or 1:10) cDNA solution equivalent to 20 ng cDNA, 2.8 µL of 3 µM primer dilutions, 10 µL PowerUp Sybr Green Kit (Thermo Fisher, Waltham, MA, USA; A25742) and 3.2 µL nuclease-free H_2_O. The samples were amplified for 40 cycles (3 s at 95 °C and for 30 s at 60 °C) after starting the reaction at 95 °C for 20 s. The melting stage to obtain the melting curves was performed at 95° for 15 s, 60° for 1 min and 95 °C for 15 s with the LightCycler^®^ 480 II (Roche, Basel, Switzerland). Data analysis was performed with the LightCycler^®^ 480 Software 1.5 (Roche, Basel, Switzerland). Ct values of the markers were referred to the corresponding Ct value of the endogenous control, 18sRNA, of respective samples and normalized to ∆Ct values of the parental HTB-41. The 2^−ΔCt^ values were further normalized against the expression levels of the parental HTB-41 and displayed as x-fold values. Primer sequences are listed in the Table S1.

#### 2.3.2. Immunofluorescence Staining

For staining upon cultivation on microscopic slides (A. Hartenstein GmbH, Würzburg, Germany; DKR0, 10 × 10 mm), the slides were first disinfected in 24-well plates with 500 µL 70% EtOH per well for 45 min, washed twice with 500 µL PBS (Thermo Fisher, Waltham, MA, US; 1976785) per well and coated with 300 µL 0.5% gelatin/well for 30 min at room temperature. Then, cells were seeded at a density of 8 × 10^3^ cells/cm^2^ for the parental HTB-41 at passage 6 or for the clones at passage 8–11 after isolation, with media changes performed every 2–3 days. Staining was accomplished on day 10 after seeding.

Cell layers were washed twice with 300 µL PBS containing Mg^2+^/Ca^2+^ (Sigma-Aldrich, St. Louis, MO, USA; D8662) following fixation and permeabilization with 300 µL pre-cooled (at −20 °C) methanol at −20 °C for 20 min. After washing twice with 300 µL PBS per well, cells were kept in PBS for 15 min for rehydratization. Then 250 µL primary antibody solutions of α-amylase, cytokeratin 5/8, vimentin or ZO-1 diluted in PBS containing 1% BSA (Sigma-Aldrich, St. Louis, MO, USA; A9647) were added over night at 4 °C (antibody list in Table S2). Cells were washed three times with 300 µL PBS before incubation with 250 µL of secondary antibody solutions (diluted in 1% BSA/PBS) at 37 °C for 1 h in the dark. For staining of the cell nuclei 250 µL DAPI solution per well (Sigma-Aldrich, St. Louis, MO, USA; D9542, 1:5000 in PBS of a 5mg/mL stock solution) was added after washing three times with 300 µL PBS for 10 min at room temperature in the dark. After removal of the DAPI solution, cells were washed three times with 300 µL PBS and embedded with 9 µL Everbright Mounting Medium (Biotium, Hayward, CA, USA; 23003) per slide. Immunofluorescence images were recorded with an Olympus IX83 microscope equipped with a SOLA-SM LED Light Engine (Lumencor, Beaverton, OR, US), controlled by CellSens Software. Processing of the images was performed with OlyVIA Software 2.9 (Olympus, Vienna, Austria).

### 2.4. Transport Studies with Ferritin

Cells of clone B2 were cultured in Transwell^®^ models as described above at passage 25–29 after single cell cloning. Transport studies were performed on day 15 after seeding. TR146 cells were used for transport studies from passage 13–20. Experiments were performed on day 29 after seeding. Cultivation and seeding procedure of TR146 cells in Transwell^®^ models was described in detail previously [22]. On the day of transport studies TEER was measured in both models as described above in the respective cultivation media, washed twice on the apical and basolateral side with 300 µL or 900 µL basal McCoy media (McCoy’s 5A media without supplements) for clone B2 or Hank´s Balanced Salt Solution (HBSS; Sigma-Aldrich; St. Louis, MO, USA; H6648) for TR146. Subsequently, TEER was measured in basal McCoy media or HBSS after equilibration for 30 min at room temperature prior to the transport studies. Human ferritin (Aviva Systems Biology, San Diego, CA, USA; OPSA10506) was then applied at a concentration of 300 ng/mL and 1000 ng/mL in the respective media—basal McCoy media or HBSS—by exchange of the total apical (A) or basolateral (B) medium for transport studies from the apical to the basolateral (A/B) compartment or from the basolateral to the apical (B/A) side.

After incubation for 24 h at 37 °C, samples from the apical and basolateral side were collected and stored at −20 °C until analysis with ELISA. In addition, cells were lysed for RNA isolation as described above to determine the expression of the transferrin receptor (TfR) by qPCR. For analysis of ferritin in the media samples with ELISA, high-binding microtiter plates (Greiner Bio-One GmbH, Kremsmünster, Austria; 655094, 96-well) were coated with 100 µL/well of 2 µg/mL ferritin antibody (East Coast Bio, North Berwick, ME, USA; HM304) dissolved in PBS sealed with an aluminum foil over night at 4 °C under gentle orbital shaking conditions. On the next day, wells were washed three times with 100 µL/well blocking buffer (PBS containing 1% bovine serum albumin (BSA; Carl Roth, Karlsruhe, Germany; 0163.2) 0.1% Tween 20 (Sigma-Aldrich, St. Louis, MO, USA; P7949). Afterwards 300 µL/well of blocking buffer was applied and incubated on an orbital plate shaker at room temperature for 1.5 h. A washing step with 300 µL blocking buffer was performed immediately prior to filling of 100 µL sample in duplicates. Stock solutions of the apical and basolateral side and used stock concentrations of ferritin were additionally diluted 1:100 in basal McCoy media or HBSS prior to addition.

A standard curve with ferritin was freshly prepared for each plate as a threefold dilution series (0, 0.03, 0.1, 0.3, 1, 3, 10, 30, 100, 300, 1000 ng/mL) in blocking buffer. HBSS and basal McCoy media without ferritin were used for background control. After incubation at room temperature on the orbital shaker sealed with aluminum foil for one hour, the wells were washed with 100 µL blocking buffer for three times. For measurement, 100 µL SuperSignal^TM^ ELISA Pico Substrate (Thermo Fisher, Waltham, MA, USA; 37070) consisting of 50:50 Super Signal ELISA Pico Luminal/Enhancer Solution and SuperSignal ELISA Pico Stable Peroxide Solution, was added per well. The plate was sealed with aluminum foil and incubated in the dark at room temperature for up to 15 min prior to the measurement of the chemiluminescent signal with the EnSpire Multimode Plate Reader (PerkinElmer, Waltham, MA, USA). The permeated concentration of ferritin was calculated as apparent permeability (P_app_) using the formula below with c_rec_ as the measured concentration in the receiving chamber (ng/mL), V_rec_ as the volume of the receiving compartment (mL), dt as the time period of the transport in (s), A giving the surface area of the insert membrane (cm^2^) and c_0_ as the measured concentration of the applied stock solution (ng/mL).

(1) $$P_{\mathrm{app}}=\;\frac{c_{\mathrm{rec}}\times V_{\mathrm{rec}}}{\mathrm{dt}\times A\times c_{0}}$$

### 2.5. Statistical Analysis

Results are shown as mean ± SEM, if not otherwise indicated. Statistical analysis was performed with SigmaPlot 14.0 (Systat, Jose, CA, USA) as Student’s *t*-test, one-way ANOVA or two-way ANOVA with Holm–Sidak or Dunn´s method as post-hoc tests with α = 0.05, * *p* < 0.05, ** *p* < 0.01, *** *p* < 0.001. Heatmap was illustrated with Qlucore Omics Explorer 3.6 (Qlucore, Lund, Sweden).

## 3. Results

### 3.1. Establishment of Salivary Gland Epithelium Barrier Models

**Figure 1:**
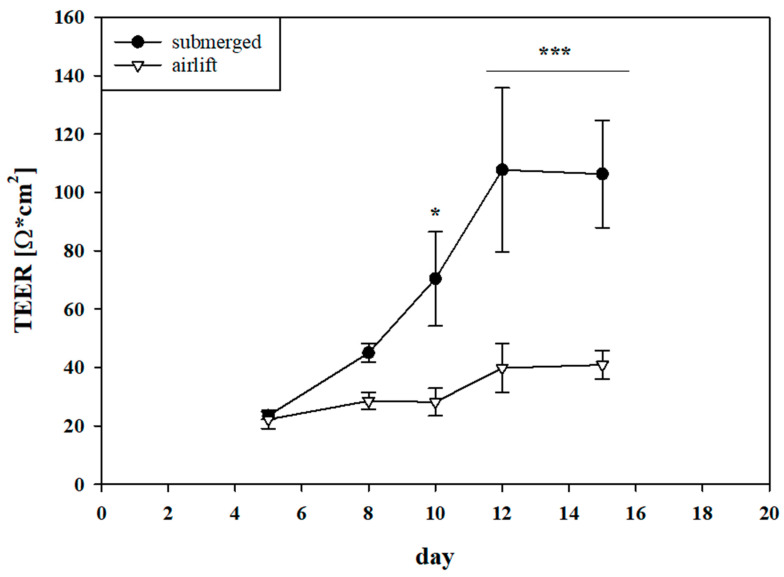
TEER progression (Ω × cm^2^) of parental cell line HTB-41 cultivated on Transwell^®^ inserts was measured as soon as cell layers reached confluency on day 5. Data are shown as mean ± SEM of three independent experiments (*n* = 5–7) cultivated in McCoy media under submerged and airlift condition. Statistical analysis was performed as two-way ANOVA followed by post-hoc Holm–Sidak test with α = 0.05, * *p* < 0.05, *** *p* < 0.001.

Monitoring of TEER values every 2–3 days until day 15 of parental HTB-41 cells cultivated on Transwell^®^ inserts either under submerged or airlift conditions showed a significant increase of TEER over time under submerged condition starting on day 10 with 70.4 ± 16.14 Ω × cm^2^ (*n* = 7, *p* < 0.05) compared to the airlift condition with 28.16 ± 4.70 Ω × cm^2^ (Figure 1). On day 12 maximum TEER of 107.72 ± 28.08 Ω × cm^2^ (*n* = 5, *p* < 0.001 compared to airlift: 39.78 ± 8.38 Ω × cm^2^) was measured under submerged condition, reaching a plateau on day 15 with TEER values of 106.31 ± 18.4 Ω × cm^2^. TEER values were calculated from measured ohmic values of 658 ± 54.04 Ω for the cells before subtraction of blank average (338 ± 6.17 Ω) and multiplication with the surface area of 0.336 cm^2^ (*n* = 5, *p* < 0.001 compared to airlift: 40.85 ± 4.89 Ω × cm^2^ or 464.25 ± 12.32 Ω). Morphological characterization by hematoxylin and eosin staining of cells cultivated under both conditions confirmed confluent HTB-41 cell layers (Figure S1).

**Figure 2:**
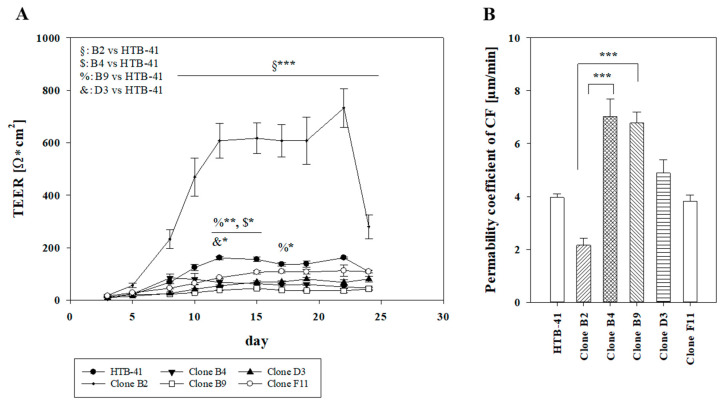
(**A**) TEER progression (Ω × cm^2^) of parental HTB-41 cell line and isolated clones B2, B4, B9, D3 and F11 over time shown as mean ± SEM from three independent experiments with *n* = 9–15 cultivated under submerged condition. Statistical analysis was performed as two-way ANOVA with post-hoc Holm–Sidak test, α = 0.05, * *p* < 0.05, ** *p* < 0.01, *** *p* < 0.001. (**B**) Permeability coefficients (µm/min) of paracellular marker carboxyfluorescein (CF) corresponding to TEER experiments shown in Figure 2A of two independent experiments (mean ± SEM, *n* = 6). Statistical analysis was performed as one-way ANOVA with post-hoc Dunn’s test, α = 0.05, *** *p* < 0.001.

Single cell cloning was performed in order to test whether cultures of isolated clones might lead to cell layers with improved paracellular barrier properties. Upon isolating five clones of HTB-41 cells, TEER experiments were performed with these clones and the parental HTB-41 cell line in the next test set-ups (Figure 2A). While the parental HTB-41 cell line reached the highest TEER values on day 12 (160.75 ± 6.31 Ω × cm^2^) and day 22 (161.28 ± 3.32 Ω × m^2^), clone B4 reached the highest resistance value on day 10 (79.68 ± 22.44 Ω × cm^2^), clone B9 on day 15 (44.62 ± 4.20 Ω × cm^2^), clone D3 on day 23 (101.53 ± 3.92 Ω × cm^2^) and clone F11 on day 22 (112.22 ± 20.84 Ω × cm^2^). Strikingly, clone B2 showed significantly higher TEER values compared to the parental HTB-41 line starting on day 8 (clone B2: 232.06 ± 36.07 Ω × cm^2^, HTB-41: 68.21 ± 5.88 Ω × cm^2^, *p* < 0.001) with a maximum 4.5-fold higher TEER value on day 22 (732.14 ± 73.37 Ω × cm^2^). The subsequent permeability studies with the paracellular marker carboxyfluorescein reflected the results of TEER experiments showing the significantly lowest permeability coefficient for clone B2 with 2.16 ± 0.26 µm/min (3.60 ± 0.43 × 10^−6^ cm/s) compared to the parental HTB-41 with 3.96 ± 0.13 µm/min. Clone B4 and clone B9 showed the highest permeability (7.01 ± 0.66 µm/min and 6.77 ± 0.42 µm/min), while clone F11 and clone D3 displayed permeability coefficient values of 3.81 ± 0.24 µm/min and 4.88 ± 0.51 µm/min, confirming the ranking of TEER values (Figure 2B). Addition of 100 nM hydrocortisone and 10 µM retinoic acid as supplements known for their barrier inducing properties to the growth media led to a significantly lower TEER value showing a peak on day 10 (hydrocortisone: 245.06 ± 15.46 Ω × cm^2^, retinoic acid: 81.76 ± 25.65 Ω × cm^2^) (Figure S2).

### 3.2. Salivary Gland Epithelium Marker Characterization of HTB-41 Clones

**Figure 3:**
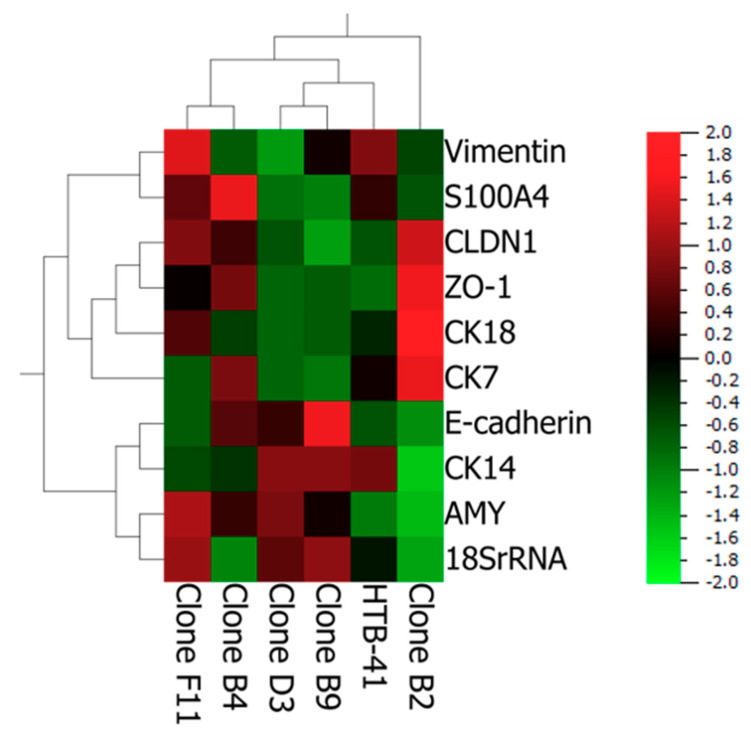
Heatmap showing qPCR results as x-fold expression values from clone B2, B4, B9, D3, and F11 normalized against expression values of the parental HTB-41 with 18SrRNA as endogenous control. Mean values of three independent experiments (*n* = 5) are displayed using a color scale, with 2 (red) indicating a high expression and −2 (green) indicating a low expression value. Mean ± SEM values and statistical analysis are shown in Table S3. AMY: α-amylase, CK: cytokeratin, CLDN1: claudin-1, E-cad: E-cadherin, Vim: vimentin, S100A4: S100 calcium-binding protein A4, ZO-1: *Zonula occludens-1*.

First, the expression of several marker molecules at the mRNA level was investigated. Data are summarized in Figure 3 as heatmap. Detailed x-fold expression values are listed in Table S3 and qPCR products for the markers are shown as agarose gels in Figure S3. Upon normalization to the parental HTB-41, clone B2 showed—as the only isolated clone—a significant downregulation of the acinar marker α-amylase (*p* < 0.001), while displaying a significant upregulation of CK18 (*p* < 0.001) as well as of ZO-1 and CLDN1 (*p* < 0.001), proteins important for tight junction formation. On the other hand, prospective markers for myoepithelial cells CK14, Vim and S100A4 were downregulated in comparison to the parental HTB-41, while showing a significant upregulation for the ductal marker CK7 (*p* < 0.001).

Isolated clone B4 showed a significant upregulation for E-cadherin, ZO-1 and CLDN1 (*p* < 0.05–*p* < 0.01) as well as the acinar marker α-amylase, while displaying a downregulation of the myoepithelial cell markers. As clone B4 also showed a significant upregulation for the ductal marker CK7 (*p* < 0.05), it was assumed for this clone to derive from two cells of acinar and ductal origin. Clone B9, D3, and F11 showed a significant downregulation for the ductal marker CK7. In addition, clone B9 and D3 also exhibited a weaker expression of vimentin and a significant downregulation of S100A4 in comparison to the parental HTB-41. Hence both clones (B9, D3) were classified to be of acinar origin. Clone F11 showed an upregulation of the myoepithelial markers vimentin and S100A4, and hence was classified to be of myoepithelial origin based on mRNA data.

**Figure 4:**
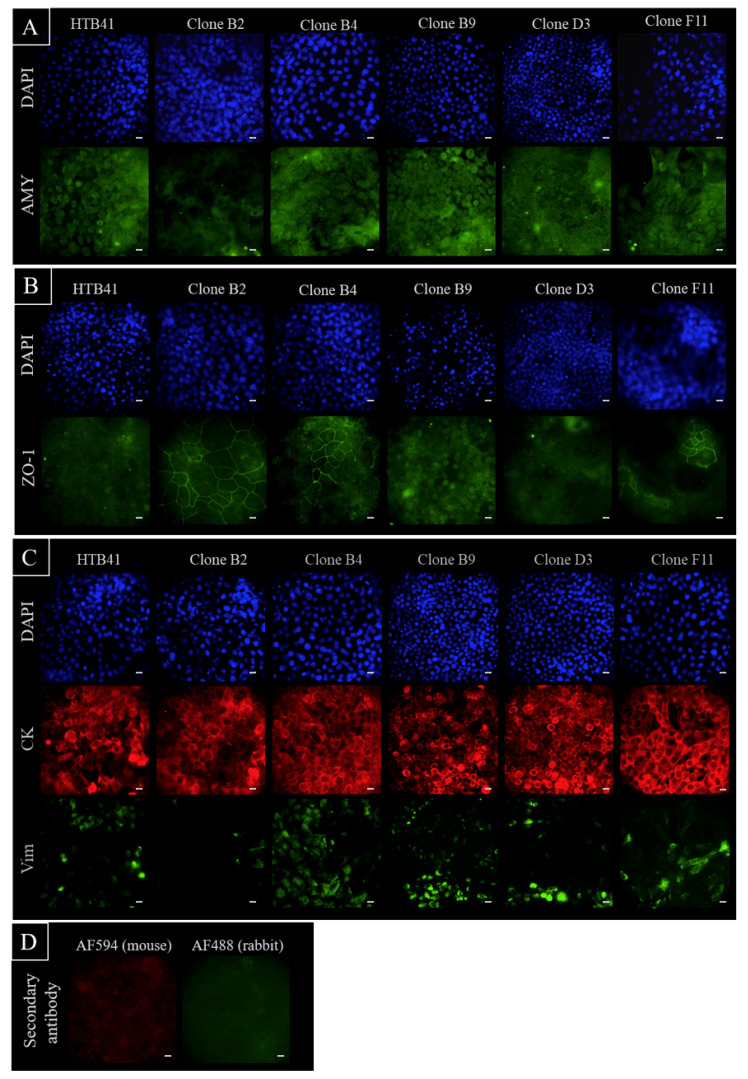
Immunofluorescence staining of parental HTB-41 cells and isolated clones B2, B4, B9, D3, and F11 seeded on microscopic slides for DAPI (blue, cell nuclei) and (**A**) α-amylase (AMY), (**B**) *Zonula occudens-1* (ZO-1), (**C**) cytokeratin 5/8 (CK) and vimentin (Vim) expression and localization analysis shown at 40× magnification. (**D**) Images of stainings at 40× magnification of controls omitting primary antibodies captured at the same settings and exposure times as stainings shown in (**A**–**C**), each white scale bar accords to 20 µm.

Immunofluorescence staining was accomplished in order to evaluate some of the qPCR results on the protein level. Images for α-amylase (Figure 4A) confirmed the qPCR results. Clone B2 showed the weakest staining for α-amylase, while depicting the highest expression of ZO-1 (Figure 4B). The positive staining of ZO-1 for clone B4 and the weak ZO-1 expression for clone F11 reflected the qPCR results as well. While all clones as well as the parental HTB-41 cells displayed a similar expression of cytokeratin 5/8, the expression of vimentin differed distinctly, clone B2 showed the weakest staining of all clones (Figure 4C). Immunofluorescence staining of clone B2, B4, B9, and D3 for ZO-1 after cultivation on Transwell^®^ inserts confirmed the presence of ZO-1 on cell-cell boarders (data not shown).

### 3.3. Transport Studies with Ferritin

**Figure 5:**
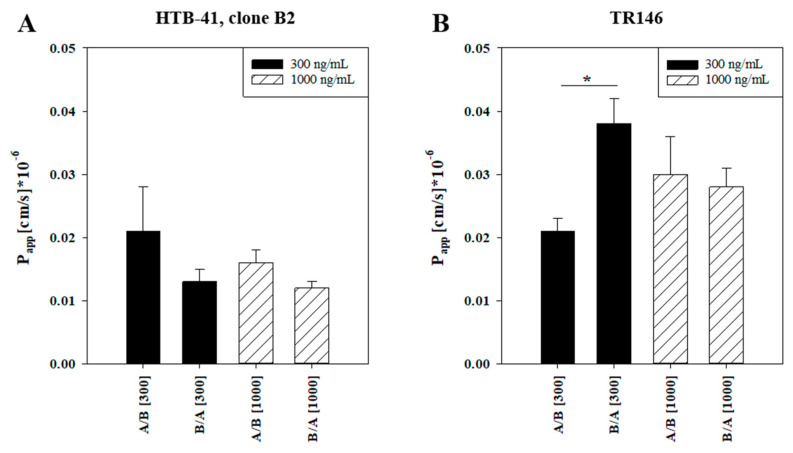
Apparent permeability coefficients P_app_ for 300 ng/mL or 1000 ng/mL ferritin applied on the apical side (transport A/B) or basolateral side (transport B/A) (**A**) in the salivary gland epithelium model based on clone B2 (*n* = 10–11 from three independent experiments) or (**B**) in the oral mucosa epithelial model based on TR146 (*n* = 10–16 from three independent experiments). Data are displayed as mean ± SEM, statistical analysis was performed as one-way ANOVA with post-hoc Dunn’s test, α = 0.05, * *p* < 0.05.

The optimized barrier model with the highest paracellular barrier based on clone B2 was used to investigate the transport of the biomarker ferritin across a salivary gland epithelium model. Ferritin was applied at two different concentrations (300 and 1000 ng/mL) either at the apical or the basolateral compartment. After 24 h the calculated apparent permeability P_app_ showed a higher, but not statistically significant permeation of ferritin from the apical (blood) to the basolateral (saliva) side (A/B) in comparison to the corresponding transport from the basolateral to the apical side (B/A). In detail, P_app_ for 300 ng/mL ferritin was 0.021 ± 0.007 × 10^−6^ cm/s (A/B) versus 0.013 ± 0.002 × 10^−6^ cm/s (B/A), and for 1000 ng/mL ferritin P_app_ was 0.016 ± 0.002 × 10^−6^ cm/s (A/B) versus 0.012 ± 0.001 × 10^−6^ cm/s (B/A) (Figure 5A). Results were compared with the transport of ferritin across an oral mucosa epithelium model based on cell line TR146 (Figure 5B). Data showed in the TR146 model a significantly increased transport of ferritin from the basolateral (in this model the blood side) to the apical (saliva) side at 300 ng/mL with P_app_ of 0.038 ± 0.004 × 10^−6^ cm/s (B/A) versus 0.021 ± 0.002 × 10^−6^ cm/s (A/B). For 1000 ng/mL ferritin, the permeability was similar in both directions (P_app_ A/B: 0.030 ± 0.006 × 10^−6^ cm/s; P_app_ B/A: 0.028 ± 0.03 × 10^−6^ cm/s). In summary, it was shown that the novel salivary gland epithelium model based on clone B2 was suitable to study the transport of the salivary biomarker ferritin. Although the differences of the transport of ferritin from A/B to B/A across HTB-41 clone B2 were not significant, the same trend for enhanced ferritin transport in the direction from the blood to the saliva compartment was found in the oral mucosa epithelium model.

## 4. Discussion

Several transport systems are present in salivary glands epithelia indicating that active transport processes take place between blood and saliva. To investigate these transport processes, we aimed to establish a human submandibular salivary gland in vitro model. As only few human submandibular salivary gland models have been published, varying significantly in TEER values, we decided to develop a standardizable model with distinct paracellular barrier properties. We selected to test cell line HTB-41, one of few available cell lines of the human submandibular gland. Until now, only few studies were published describing cell line HTB-41, and no data about its paracellular barrier were reported [29,30,31].

First Transwell^®^ experiments with HTB-41 proved the ability of HTB-41 to form confluent layers on Transwell^®^ inserts under submerged conditions. Haematoxylin and eosin staining confirmed the morphology of a continuous cell layer. TEER values indicated the formation of a significant paracellular barrier sufficient for subsequent transport studies.

However, single cell isolation experiments were performed to obtain single clones with even more distinct barrier properties. It is known that cancer cells can exhibit inhomogeneity, this was already described in the late 1950s [32]. Isolation of single clones from cancer cell lines is a very well established method and was already performed in the 1980s [33,34]. For characterization of the isolated HTB-41 clones, we first tested their ability to form a paracellular barrier as a distinguishable, functional characteristic. As the only isolated clone, clone B2 showed a stronger barrier than the parental cell line. Other reports confirmed the ability of cell lines derived from the ductal epithelium of the submandibular gland to form distinct paracellular tightness. For example, tumor derived cell line HSG (human submandibular gland) reached TEER values between 50 and 417 Ω × cm^2^ depending on the type of Transwell^®^ inserts, or HSDEC (human submandibular gland ductal epithelial cells), an immortalized cell line, achieved TEER values of about 1700 Ω × cm^2^ [35,36].

According to these high TEER values, it was assumed that clone B2 might be also of ductal origin. While the integrity of the paracellular barrier could be used as a first characteristic for cell type classification, it is crucial to further characterize the clones at the mRNA and protein level applying several different markers for each cell type. In this context, Azevedo et al. (2008) highlighted the importance of using several markers for one cell type, as the expression of multiple cytokeratins was not restricted to one cell type, while the diverse patterns of the expressed cytokeratins enabled a proper classification [14]. With regard to the used markers in the current study, vimentin was described in several sources as a myoepithelial marker, whereas S100 was either classified as a myoepithelial or ductal cell marker [13]. In our study, S100A4 seemed to be a marker for myoepithelial cells as its expression was upregulated in the myoepithelial clone F11. In this regard, cell type marker expression studies showed that clone B2 revealed the weakest expression of the acinar marker α-amylase as well as of the myoepithelial markers. CK18, a marker often confirmed in acinar cells, but also found in ductal cells as well [27], was significantly upregulated at the mRNA level in this clone. However, immunofluorescence staining for CK18 showed no clear difference between clone B2 and parental HTB-41 (data not shown) corresponding to the fact that CK18 was found in acinar as well as ductal cells previously. The significant upregulation of tight junction proteins claudin-1 and ZO-1 corresponded well with high TEER values and the formation of a distinct paracellular barrier as well as the distinct higher expression of the presumably ductal marker CK7 confirmed the assumption for clone B2 to be of ductal origin. The separate clustering of clone B2 in the heatmap upon comparison of marker expression to the parental cell line and other isolated clones (Figure 3) underlined the distinct characteristics for clone B2.

The remaining four isolated clones B4, B9, D3, and F11 were classified being from acinar or myoepithelial cell origin based on the TEER data and marker expression. While clone B4 expressed significantly more α-amylase than the parental cell line, the ductal cell marker (CK7) was also upregulated, but both myoepithelial markers were downregulated. Hence, clone B4 was classified to be a mixture of acinar and ductal cells. In clone B9 and clone D3 myoepithelial markers vimentin, S1004 as well as the ductal marker CK7 were downregulated, whereas acinar marker α-amylase was upregulated. Thus, both clones were classified as acinar cells. Clone F11 showed as the only clone an upregulation of vimentin and a weak paracellular barrier. Therefore, clone F11 was classified as being of myoepithelial origin.

To further investigate the differences between the parental HTB-41 cell line and the isolated clone B2, flow cytometry experiments were performed with α-amylase, cytokeratin 5/8, vimentin, and ZO-1 (Table S4). Interestingly, two distinct cell populations with differing vimentin staining behavior were found for the parental cell line, confirming the heterogeneity of the parental cell line. On the contrary, clone B2 consisted of only one vimentin population. Additionally, the majority of clone B2 showed a strong positive staining (91.5%) for ZO-1, compared to the parental HTB-41 cell population (35.7%). The intensity of the ZO-1 staining also correlated with the granularity of the cell population (Figure S4).

Only few studies based on primary epithelial salivary gland cells have been published until now, perhaps because the accessibility of biopsy samples of salivary glands is limited. In 2005, Tran et al. described a primary cell model of the human submandibular gland exhibiting TEER values of 250–300 Ω × cm^2^, followed by a study of Hegyesi et al. in 2015 showing that an adaption of the protocol led to TEER values of 622 ± 117 Ω × cm^2^ (mean ± SEM) on day seven of cultivation [19,25]. Further studies using human primary models of the submandibular gland focused on the characterization of their morphology or on their application for enzymatic activity assays [37,38,39].

In this context, clone B2 was chosen for the transport experiments as this clone showed the tightest paracellular barrier reaching TEER values of 732 ± 73 Ω × cm^2^ being in a similar TEER range as the optimized primary cell model of Hegyesi et al. (2015) [19]. In biomarker research, transport studies across salivary gland models could be used to verify the validity of a salivary biomarker as such. Understanding the transport of a salivary biomarker across the BSB could provide the causal link between the saliva and the blood concentrations of the respective biomarker. In order to test the suitability of the optimized in vitro model based on clone B2 for such transport studies, we selected to study the transport of ferritin, a serum biomarker associated with iron storage [40]. Elevated serum levels of ferritin were measured in patients with diabetes, periodontitis or anemia, correlating with salivary ferritin levels [41,42]. For example, Guo et al. (2018) measured ferritin concentrations of 196.2 ng/mL in serum and 6.50 ng/mL in saliva (*n* = 22, median) in healthy individuals while subjects with chronic periodontitis showed elevated ferritin levels of 265.1 ng/mL in serum and 16.74 ng/mL in saliva (*n* = 22, median) [43]. While ferritin levels greater than 1000 ng/mL in serum was described as an overall marker for diseases [44], levels lower than 30 ng/mL were postulated to be an indicator for iron deficiency [45]. Decreased serum ferritin levels were also correlated with a higher risk for cardiovascular diseases in patients with type 2 diabetes [46].

To study the transport of ferritin in our models, 300 or 1000 ng/mL were applied either on the apical (= blood compartment) or basolateral (= saliva compartment) side. After 24 h incubation sufficient amounts of ferritin were detected on each opposite side, underlining the suitability of the established model for transport studies with high-molecular weight biomarkers. Although no significant concentration or permeation direction dependent effects were found in the salivary gland model, the tendency to permeate faster into the saliva compartment was detected for ferritin. Corresponding to this, ferritin (300 ng/mL applied) permeated significantly faster from the blood (basolateral compartment) to the saliva side compared to the other direction in the oral mucosa model. Studies across both BSB models indicated the general tendency for ferritin to be transported faster from blood to saliva and suggested the suitability of ferritin as salivary biomarker. In 2009, the transport mechanism of ferritin mediated by the endocytosing cell-surface receptor transferrin receptor-1 (TfR-1) was described comprehensively [47]. With TfR-1 being ubiquitously expressed, it was hypothesized that the postulated transport mechanism of ferritin via TfR-1 across the blood–brain barrier might also work at the BSB. Therefore, we examined the expression of the transferrin receptor (TfR) upon application of ferritin and compared the expression to untreated cells by qPCR. Expression of TfR of ferritin-treated B2 cells was significantly decreased in both directions and applied concentrations in comparison to untreated samples (Figure S5A). On the contrary, in the oral mucosa model treatment with ferritin for 24 h upregulated TfR expression in comparison to the untreated control (Figure S5B). However, no regulation of TfR was found on the protein level in both models (Figure S5C,D). Different transport data and opposed regulation of TfR in both models indicate the need and importance to involve models of several parts of the BSB for comprehensive biomarker studies. With regard to the transport of ferritin across the BSB, further studies are needed to elucidate the mechanisms and regulation of ferritin transport in detail.

## 5. Conclusions

The ability ofthe human submandibular salivary gland cell line HTB-41 to form distinct paracellular barrier properties was described for the first time. Isolation of single clones of the heterogeneous cell line HTB-41 resulted in five clones with distinct different properties and cell type characteristics (acinar, ductal, myoepithelial), and led to a clone with significantly increased barrier properties compared to the parental cell line. Transport studies with the potential salivary biomarker ferritin showed the applicability of the optimized model for biomarker studies.

## Figures and Tables

**Table 1:** Overview of the markers used for characterization of the salivary gland cell types.

	Marker for	References
α-Amylase	Acinar cells	Nagao et al. (2012) [11]
		Namboodiripad et al. (2014) [12]
CK7	Ductal cells	Zhu et al. (2005) [13]
		Dreager et al. (1991) [28]
	Intercalated, striated ducts	Azevedo et al. (2008) [14]
CK14	Myoepithelial cells	Nagao et al. (2012) [11]
		Azevedo et al. (2008) [14]
CK18	Acinar and ductal cells	Namboodiripad et al. (2014) [12]
		Azevedo et al. (2008) [14]
		Kusama et al. (2000) [27]
CLDN1	SMG	Baker et al. (2016) [24]
	Intercalated/striated duct cells, weak in serous cells	Maria et al. (2008) [26]
E-cadherin	SMG	Baker et al. (2016) [24]
		Tran et al. (2005) [25]
S100	Myoepithelial cells, Intercalated duct cells	Zhu et al. (2005) [13]
Vimentin	Myoepithelial cells	Nagao et al. (2012) [11]
		Namboodiripad et al. (2014) [12]
		Zhu et al. (2005) [13]
ZO-1	SMG	Baker et al. (2016) [24]
		Tran et al. (2005) [25]
		Maria et al. (2008) [26]

CK: cytokeratin, CLDN1: claudin-1, S100A4: S100 calcium-binding protein A4, SMG: submandibular gland, ZO-1: *Zonula occludens-1*.

## Supplementary Materials

Supplementary materials can be found at https://www.mdpi.com/2227-9059/8/9/302/s1. Figure S1: Hematoxylin–Eosin staining (HE) of HTB-41 upon cultivation under airlift and submerged conditions on 24-well Transwell^®^ inserts; Figure S2: TEER values [Ω × cm^2^] over time of clone B2 as mean ± SEM under submerged condition, supplemented with 100 nM hydrocortisone (HC) and 10 µM retinoic acid (RA); Figure S3: Agarose gels after quantitative real-time PCR showing the tested markers of HTB-41 and Clone B2, B4, B9, D3 and F11 applied against a 50 bp ladder; Figure S4: Results of flow cytometry of parental HTB-41 and clone B2 cells; Figure S5. Expression of the transferrin receptor (TfR) at the mRNA level of the salivary gland model based on clone B2 and of the oral mucosa model based on TR146 cells; Table S1: Primer sequences of applied markers for qPCR; Table S2: Solutions used for immunofluorescence staining; Table S3: Statistical analysis of qPCR results of cell-type specific markers of isolated clones B2, B4, B9, D3, and F11 normalized to parental HTB-41; Table S4: Solutions used for flow cytometry with the applied dilution in 50 µL cell suspension.
