# Supplementary material for: An In Vitro Barrier Model of the Human Submandibular Salivary Gland Epithelium Based on a Single Cell Clone of Cell Line HTB-41: Establishment and Application for Biomarker Transport Studies
Source: Biomedicines. 2020 Aug 23;8(9):302. doi: 10.3390/biomedicines8090302 (PMC7555419; doi:10.3390/biomedicines8090302)
Supplement: Supplementary file 1 [file biomedicines-08-00302-s001.pdf]

Supplementary Method

Haematoxylin and Eosin staining of Transwell® inserts

For Haematoxylin and Eosin stainings HTB-41 grown on Transwell® inserts were washed twice with PBS containing Ca^{2+} and Mg^{2+} (Sigma-Aldrich, St. Louis, MO, US; D8662) and fixed with 200 µL 4 % paraformaldehyde (PFA) solution for at least 2 hours at room temperature. Until staining, inserts were stored at 4°C in 50 mL tubes covered by PBS. The procedure for the staining was described in detail previously[1].

Agarose gel

For the visualization of the quantitative real-time PCR results, the products were diluted 1:6 with 6x DNA loading dye (Fermentas, Vilnius, Litauen; R0611) and separated on a 2 % agarose gel containing 0.08 % SYBR Safe® (Invitrogen, Carlsbad, CA, US; 33102) in 1x Tris-Borate-EDTA (TBE) buffer in a 34-well chamber system (Peglab, Hong Kong, China) for 1 h and 40 min at 120 Volt and 120 mA. Images were taken with the UVP Biospectrum 310 imaging System.

Flow Cytometry

For flow cytometric analysis, HTB-41 cells at passage 4 and clone B2 at passage 40 upon isolation cultivated in T25 flasks in McCoy media were trypsinized as described in the material and method section in the main manuscript. For the staining, cell suspensions containing $1.5*10^6$ cells were centrifugated for 5 min at 300 g at room temperature. Supernatant was aspirated and cells were fixed and permeabilized with 400 µL of the fixation and permeabilization solution provided in the BD Cytofix/Cytoperm™ kit (BD Biosciences, Franklin Lakes, NJ, US; 554714) for 20 min at 4 °C. Fixed cells were centrifuged at 300 g for 5 min at room temperature and washed with 250 µL

PBS, following a centrifugation step for 5 min at 300 g and room temperature. Prior to aliquoting the cells for antibody staining, the cell pellet was resuspended in 250 µL 1:10 diluted Perm/Wash solution provided in the BD Cytofix/Cytoperm^{TM} kit. 1-5 µL primary antibody solutions with α-amylase, cytokeratin5/8, lactoferrin, vimentin and ZO-1 (see supplementary Table S4) was added to 50 µL cell suspension containing 50,000 cells and incubated for 30 min in the dark at 4°C. Each sample was washed with 3 mL PBS, following another washing step with 3 mL 1:10 diluted Perm/Wash buffer, with centrifugation steps of 5 min at 300g at room temperature in-between. Cell pellets were reconstituted in 50 µL 1:10 diluted Perm/Wash buffer and incubated with 1 µL secondary antibody solution for 30 min in the dark at 4 °C (Table S4). After washing, first with 3 mL PBS andthen with 3 mL 1:10 BD Perm/Wash solution with centrifugation steps of 5 min 300g at room temperature in-between, the pellets were reconstituted in 300 µL FACS buffer (PBS containing 2 mM EDTA, 1 % BSA) for analysis with the flow cytometer. For data acquisition 10,000 cells were analysed with the CytoFLEX Flow Analyzer 2L4 (Beckman Coulter, Brea, CA, US) using the software Cytoflex. To eliminate cell debris, thresholds of forward scatter (FSC) and side scatter (SCC) were set with cells without antibody solutions. For distinction between specific staining and background signal of secondary antibody solutions, threshold was set with cell suspension containing both secondary antibody solutions, recorded with the FITC (488 nm excitation, 525/50 nm emission) and PC5.5 (561 nm excitation, 710/50 nm emission) channel. Compensation matrix was adjusted to eliminate spillover between the channels FITC and PC5.5. Data analysis and illustration was performed with the Kaluza software (2.1, Beckman Coulter, Brea, CA, US).

Western Blot

The isolated clone B2 from the submandibular gland cell line HTB41 was seeded at passage 35 and 48 upon isolation at a cell concentration of 8 x 10^4 cells/cm^2 on 6-well Transwell inserts (Greiner Bio-One GmbH, Kremsmünster, Austria; 657641) in 2 mL apical McCoy media, provided with 3 mL McCoy media in the basolateral compartment. Media change of the apical and basolateral side was performed every 2-3 days until lysis on day 15 or 17. Similarly, the oral mucosa cell line TR146 was seeded on 6-well Transwell inserts at passage 26 and 32 at a cell concentration of 9.33 x 10^3/cm^2 in 2 mL DMEM media, with 3 mL DMEM media provided on the basolateral side. Cultivation of TR146 on Transwell inserts was described in detail previously[2]. In brief, cells were switched to airlift cultivation as soon as the cells reached confluency and DMEM media was additionally supplemented with Human Keratinocytes Growth Supplement (HKGS, Thermo Fisher, Waltham, MA, US; S0015). Media change was performed every 2-3 days basolaterally and cells were lysed on day 30-31 for western blotting. Procedure for western blotting was described in detail previously by Gerhartl et al. (2020)[3]. In brief, cells were lysed with 50 µL RIPA buffer supplemented with PhosphoSTOP and ULTRA protease inhibitor cocktail (Roche, Basel, Switzerland; 04906837001 and 0589297000). Protein concentration was determined with the BCA assay prior to loading 20 µg protein on 10 % SDS gels. Primary antibody for transferrin receptor (TfR; Thermo Fisher, Waltham, MA, US; 13-6800, mouse, 1:500) and anti-mouse IgG, HRP-linked secondary antibody (Cell signaling, Danvers , MA, US; 7076, horse, 1:5000) were diluted in 5 % dry milk. HRP-linked antibody for ß-actin (Sigma-Aldrich, St. Louis, MO, US; A3854, mouse) was applied 1:20,000 in 5 % dry milk. Incubation in antibody solutions and washing steps are described in detail by Neuhaus et al. (2012)[4]. Images were captured with the ChemiDoc Touch Imaging System (Bio-Rad Laboratories, Hercules, CA, US) and analysis was performed with the ImageLab software (5.2.1, Bio-Rad Laboratories, Hercules, CA, US).

Supplementary Figures

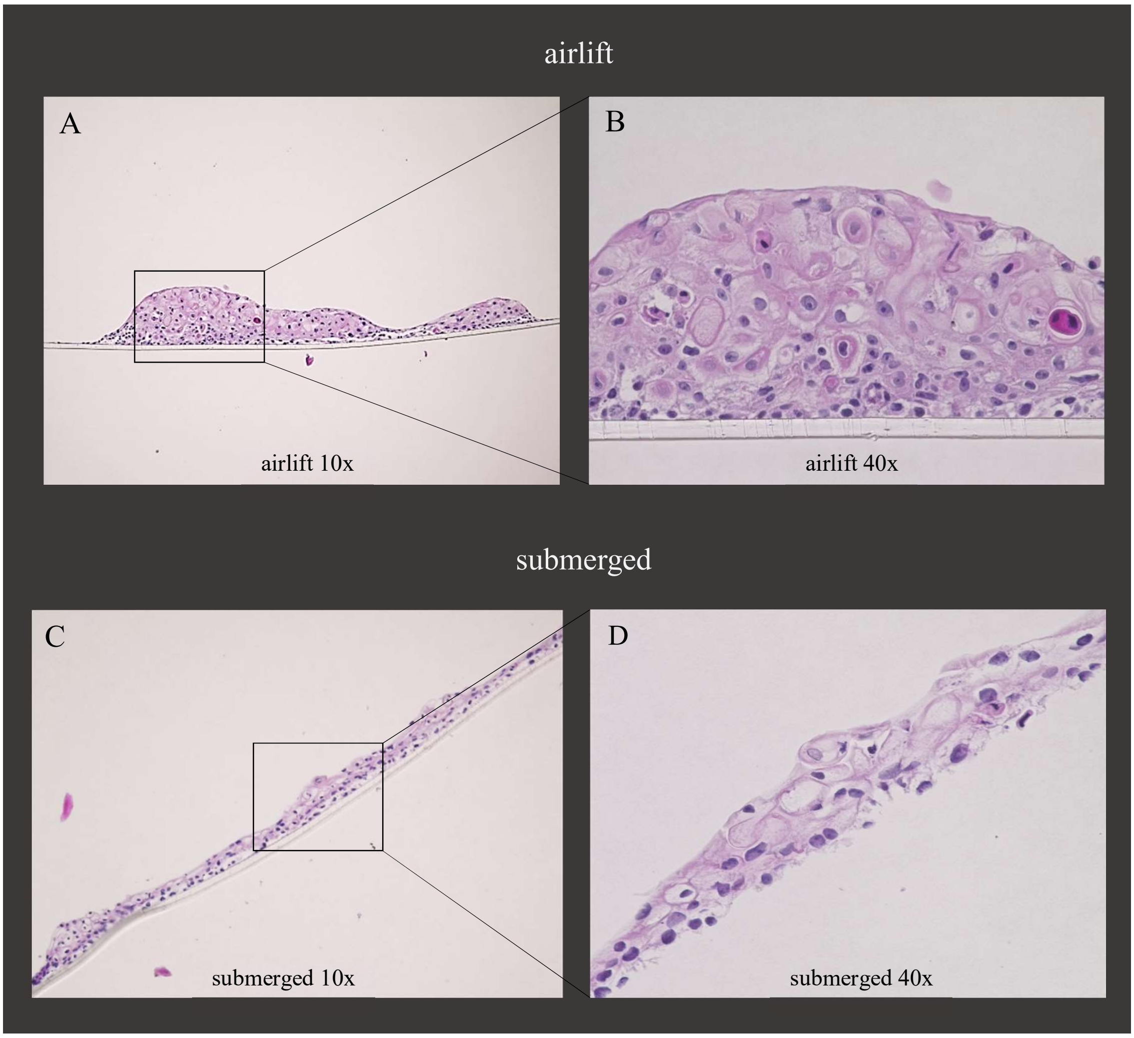

Figure S1. Hematoxylin-Eosin staining (HE) of HTB-41 upon cultivation **A-B** under airlift and **C-D** submerged conditions on 24-well Transwell® insert at 10x (A, C) magnification and 40x (B, D) magnification.

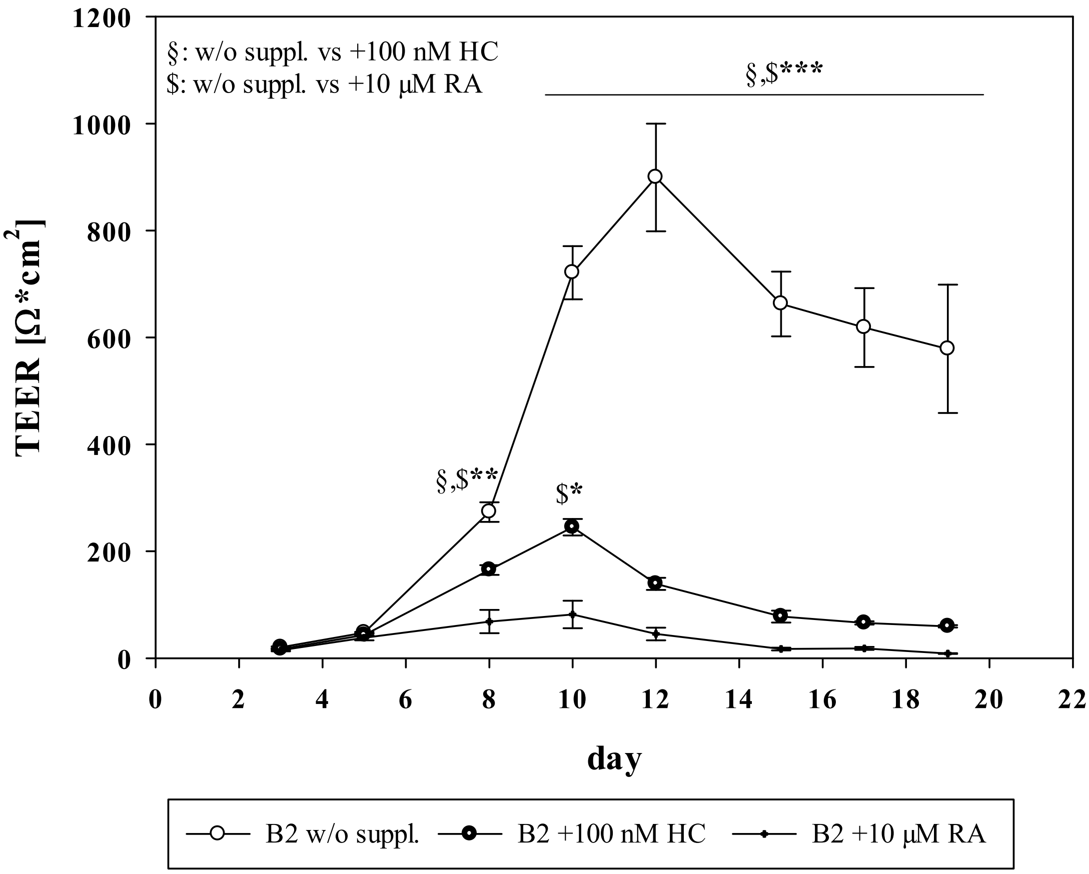

Figure S2. TEER values [Ω x cm^2] over time of clone B2 as mean ± SEM from two independent experiments (N=6-12) under submerged condition, supplemented with 100 nM hydrocortisone (HC) and 10 µM retinoic acid (RA). Statistical analysis was performed as two-way ANOVA and Holm-Sidak as post hoc test, $\alpha = 0.05$, $p<0.01$**, $p<0.001$***.

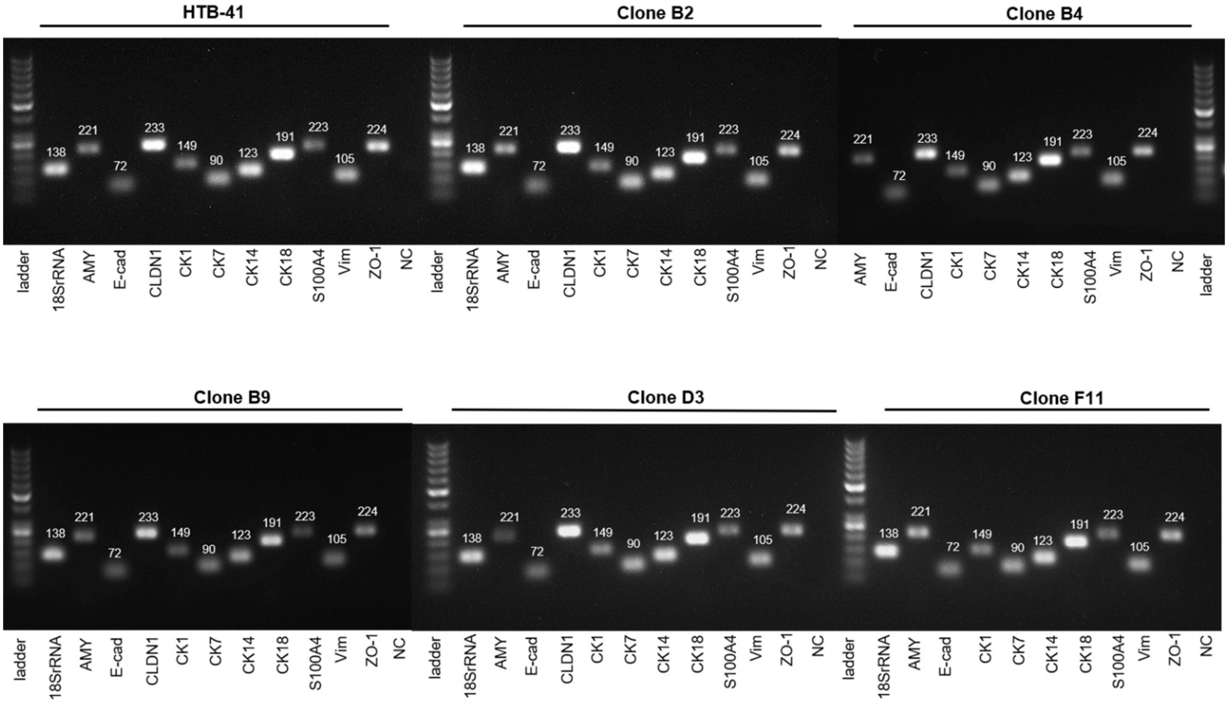

Figure S3. Agarose gels after quantitative real-time PCR showing the tested markers of HTB-41 and Clone B2, B4, B9, D3 and F11 applied against a 50 bp ladder. AMY: α-amylase, CK: cytokeratin, CLDN: claudin, E-cad: E-cadherin, NC: negative control, Vim: vimentin, S100A4: S100 calcium-binding protein A4, ZO-1: *Zonula occludens-1*

HTB-41 Clone B2

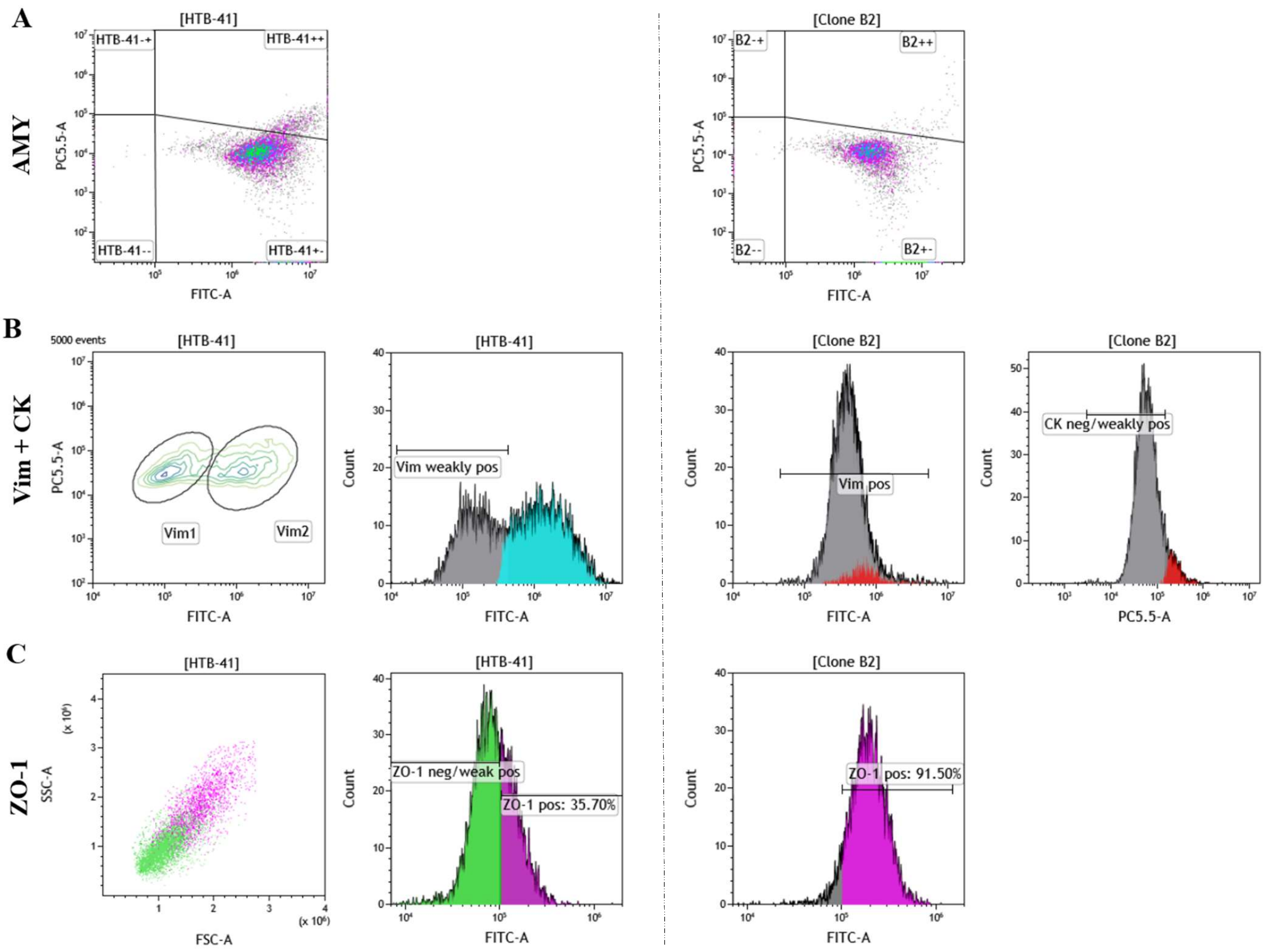

Figure S4. Results of flow cytometry of parental HTB-41 and clone B2 cells. **A** Staining of α-amylase (FITC) shown as density plot. **B** Staining for vimentin (FITC) and cytokeratin (PC5.5) shown as contour plot or histogram. Cell population of HTB-41 with a vimentin staining intensity comparable to negative control coloured in grey (vim1), cell population with a stronger staining intensity coloured in blue (vim2). Cells of clone B2 with a positive signal for vimentin are coloured in grey, cells with a positive staining for cytokeratin coloured in red. **C** Positive staining of ZO-1 in magenta, cells with a staining intensity of ZO-1 similar to negative control coloured in green. Applied antibody dilution is shown in supplementary Table S4.

While parental HTB-41 and B2 cells were all positive for α-amylase, co-staining of vimentin and cytokeratin (CK) 5/8 led to two populations with different vimentin staining intensity for the parental HTB-41 with 34.4

% showing a signal intensity of $10^{4.5-}$-$10^{5.5}$ for and 62.6 % a signal intensity of $10^{5.5-}10^{7}$ for vimentin. On the contrary, clone B2 showed a homogenous vimentin staining at a signal intensity of 10^{6-} 10^{7}. Clone B2 also showed to contain cells with a more intense cytokeratin staining (8.4 %).

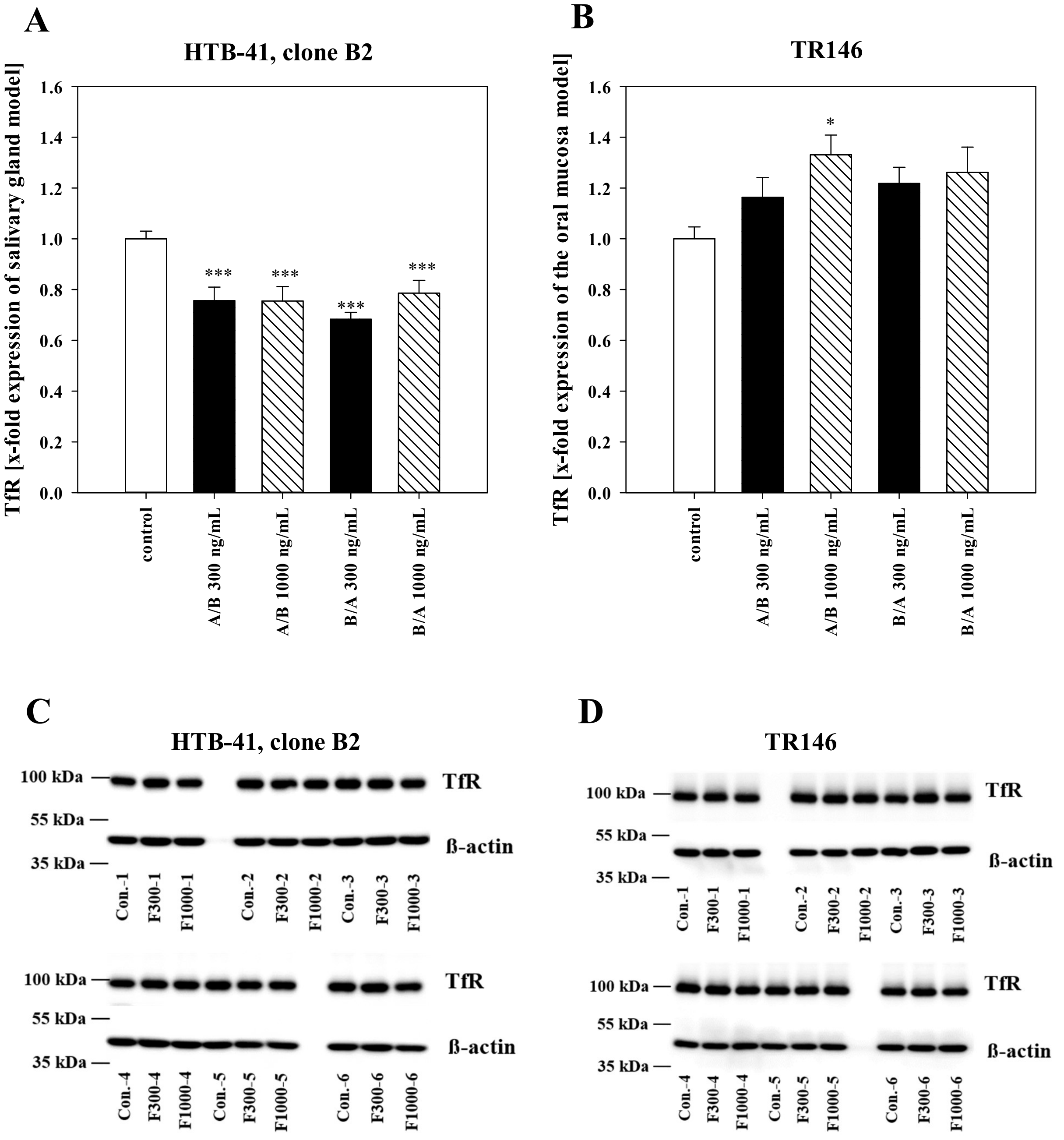

Figure S5. Expression of the transferrin receptor (TfR) at the mRNA level of the salivary gland model based on clone B2 (**A**) and of the oral mucosa model based on TR146 cells (**B**). Samples lysed after transport studies applying 300 or 1000 ng/mL ferritin on the apical (A/B) or basolateral (B/A) side for 24 h were analysed by qPCR. ΔCt values of three independent experiments were referred to corresponding 18SrRNA values and normalized to the control samples. Results shown as mean ± SEM, from three (clone B2) independent experiments with N=9-12 or five (TR146) independent experiments with N=15-30. Statistical

analysis was performed as one-way ANOVA, with post-hoc Dunn´s test, $\alpha = 0.05$, $p<0.05$*, $p<0.001$***. **C-D**: Protein expression of TfR and endogenous control (ß-actin) of the salivary gland model based on clone B2 (**C**) and of the oral mucosa model based on TR146 cells (**D**) showing control samples (con.), samples treated with 300 ng/mL ferritin (F300) or 1000 ng/mL ferritin (F1000) on the apical and basolateral side in two independent experiments (N=6). Statistical analysis was performed as Student´s t-test with $\alpha = 0.05$ upon referring to the untreated control. No significant regulation was detected after normalization to control samples either for the salivary gland model (control: 1.00 ± 0.09 (mean ± STD); 300 ng/mL ferritin: 1.02 ± 0.09, p=0.68; 1000 ng/mL ferritin: 0.97 ± 0.10, p=0.60) or the oral mucosa model (control: 1.00 ± 0.12 (mean ± STD); 300 ng/mL ferritin: 1.04 ± 0.11, p=0.63; 1000 ng/mL ferritin: 0.97 ± 0.07, p=0.67).

1. Rossi, A., Appelt-Menzel, A., Kurdyn, S., Walles, H. & Groeber, F. Generation of a Three-dimensional Full Thickness Skin Equivalent and Automated Wounding. *J. Vis. Exp.* 1–7 (2015) doi:10.3791/52576.

2. Lin, G. C. *et al.* Optimization of an oral mucosa in vitro model based on cell line TR146. *Tissue barriers* **8**, 1748459 (2020) doi:10.1080/21688370.2020.1748459.

3. Gerhartl, A. *et al.* Hydroxyethylstarch (130/0.4) tightens the blood-brain barrier in vitro. *Brain Res.* 1727, 146560 (2020) doi:10.1016/j.brainres.2019.146560.

4. Neuhaus, W. *et al.* Lung endothelial cells strengthen, but brain endothelial cells weaken barrier properties of a human alveolar epithelium cell culture model. *Differentiation* (2012) doi:10.1016/j.diff.2012.08.006.

Table S1. Primer sequences of applied markers for qPCR. AMY: α-amylase, CK: cytokeratin, CLDN: claudin, E-cad: E-cadherin, Vim: vimentin, S100A4: S100 calcium-binding protein A4, TfR: Transferrin receptor, ZO-1: *Zonula occludens-1*.

Primer	forward 5'-3'	reverse 5'-3'	Identification
18sRNA	ATGGTTCCTTTGGTCGCTCG	GAGCTCACCGGGTTGGTTTT	NM_003286.2
AMY	GAGGGGTTCAGGTCTCTCCA	TGTTCCTGCACTCACAGCAT	NM_001008218.1
CK7	CATCGAGATCGCCACCTACC	ATCACAGAGATATTCACGGCTCC	NM_005556.3
CK14	AAGACCATTGAGGACCTGAGGAA	CTCTGTCTCATACTTGGTGCGG	NM_000526.4
CK18	GAGGGCTCAGATCTTCGCAA	CCAGCTGCAGTCGTGTGATA	NM_199187.1,<br>NM_000224.2
CLDN1	CCGTTGGCATGAAGTGTATG	AAGGCAGAGAGAAGCAGCAG	NM_001307.5
E-cad	CCCGGGACAACGTTTATTAC	GCTGGCTCAAGTCAAAGTCC	NM_001317186.1,<br>NM_001317185.1,<br>NM_001317184.1,<br>NM_004360.4
S100A4	TGGTTTGGTGCTTCTGAGATGT	CCTGTTGCTGTCCAAGTTGC	NM_019554.2
TfR	AGCCCACTGTTGTATACGCT	TTTCTCAACTTTGCTGGCCC	NM_001313966.1<br>NM_001313965.1<br>NM_003234.3<br>NM_001128148.2
Vim	CGGGAGAAATTGCAGGAGGA	AAGGTCAAGACGTGCCAGAG	NM_003380.4
ZO-1	TGAGGCAGCTCACATAATGC	GGTCTCTGCTGGCTTGTTTC	NM_003257.4

Table S2. Solutions used for immunofluorescence staining.

Solution	Company	dilution
α-Amylase	Sigma-Aldrich, St. Louis, MO, US; A8273, rabbit	1:100
Cytokeratin 5/8	ProteinTech, Rosemont, IL, US; 66110-1-Ig, mouse	1:100
Vimentin	Abcam, Cambridge, UK; ab45939, rabbit	1:1000
ZO-1 (*Zonula occludens-1*)	Life technologies, Carlsbad, CA, US; 40-2300, rabbit	1:100
DAPI	Sigma-Aldrich, St. Louis, MO, US; D9542	1:5000
anti-rabbit IgG Alexa Fluor 488	Life technologies, Carlsbad, CA, US; A-21206, donkey	1:200
anti-mouse IgG Alexa Fluor 594	Life technologies, Carlsbad, CA, US; A-21203, donkey	1:200

Table S3. Statistical analysis of qPCR results of cell-type specific markers of isolated clones B2, B4, B9, D3 and F11 normalized to parental HTB-41 (three independent experiments, N=5, mean ± SEM) performed as two-way ANOVA with post-hoc Holm-Sidak against HTB-41 as control, α = 0.05, p<0.05*, p<0.01**, p<0.001***. AC: marker for acinar cells, DC: marker for ductal cells, EC: endogenous control, ME: marker for myoepithelial cells. SMG: marker for submandibular salivary gland. AMY: α-amylase, CK: cytokeratin, CLDN1: claudin-1, E-cad: E-cadherin, Vim: vimentin, S100A4: S100 calcium-binding protein A4, ZO-1: *Zonula occludens-1*.

Marker Class	Marker	HTB-41	Clone B2	Clone B4	Clone B9	Clone D3	Clone F11
EC	18SrRNA	1 ± 0.06	1 ± 0.05	1 ± 0.04	1 ± 0.07	1 ± 0.08	1 ± 0.06
SMG	E-cad	1 ± 0.04	0.77 ± 0.08	1.54 ± 0.21**	2 ± 0.30**	1.46 ± 0.14*	0.95 ± 0.11
	ZO-1	1 ± 0.06	1.92 ± 0.2***	1.61 ± 0.15**	1.05 ± 0.17	1.01 ± 0.19	1.33 ± 0.19
	CLDN1	1 ± 0.04	1.95 ± 0.2***	1.5 ± 0.19*	0.7 ± 0.08	1 ± 0.08	1.73 ± 0.15**
DC	CK7	1 ± 0.05	1.82 ± 0.14***	1.39 ± 0.09*	0.37 ± 0.03**	0.44 ± 0.03**	0.5 ± 0.03*
DC/ME	S100A4	1 ± 0.05	0.5 ± 0.06**	1.64 ± 0.19**	0.3 ± 0.04***	0.36 ± 0.06**	1.18 ± 0.09
ME	CK14	1 ± 0.03	0.55 ± 0.03*	0.77 ± 0.04	1.02 ± 0.06	1.02 ± 0.07	0.74 ± 0.04
	Vim	1 ± 0.03	0.66 ± 0.04	0.61 ± 0.05	0.81 ± 0.07	0.49 ± 0.06*	1.15 ± 0.21
AC	AMY1A	1 ± 0.02	0.51 ± 0.05**	2.4 ± 0.13***	2.18 ± 0.10***	2.88 ± 0.30***	3.25 ± 0.29***
	CK18	1 ± 0.04	1.73 ± 0.08***	0.92 ± 0.06	0.84 ± 0.07	0.81 ± 0.10	1.27 ± 0.05

Table S4. Solutions used for flow cytometry with the applied dilution in 50 µL cell suspension.

Solution	Company	dilution
α-Amylase	Sigma-Aldrich, St. Louis, MO, US; A8273, rabbit	1:10
Cytokeratin 5/8	ProteinTech, Rosemont, IL, US; 66110-1-Ig, mouse	1:10
Vimentin	Abcam, Cambridge, UK; ab45939, rabbit	1:50
ZO-1 (*Zonula occludens-1*)	Life technologies, Carlsbad, CA, US; 40-2300, rabbit	1:12.5
anti-rabbit IgG Alexa Fluor 488	Life technologies, Carlsbad, CA, US; A-21206, donkey	1:50
anti-mouse IgG Alexa Fluor 594	Life technologies, Carlsbad, CA, US; A-21203, donkey	1:50
